# Outcomes and Mechanisms of Change of the Strengthening Families Program in a Clinical Sample of Children and Their Families in Austria

**DOI:** 10.3390/ijerph19031074

**Published:** 2022-01-19

**Authors:** Elisabeth Stefanek, Tanja Bleis, Markus Schwab, Georg Spiel

**Affiliations:** 1 Pro Mente Forschung (Pro Mente Research), 1050 Vienna, Austria; markus.schwab@promente-forschung.at (M.S.); georg.spiel@promente-kijufa.at (G.S.); 2 Pro Mente: Kinder Jugend Familie (Pro Mente: Children Youth Family), 9020 Klagenfurt, Austria; tanja.bleis@promente-kijufa.at

**Keywords:** children’s mental health, family support, parenting behavior

## Abstract

Family context and parenting behavior have the greatest influence on children’s mental health and well-being, and interventions that take the whole family system into account are promising. This study aims to evaluate the outcomes, i.e., family strength, parenting behavior, and child behavior, of the Strengthening Families Program (SFP), developed by Kumpfer which was implemented in an outpatient clinic of a community-based non-governmental organization in Austria between 2012 and 2018. Furthermore, the program’s mechanism of change as formulated by the program authors (i.e., to what extent parenting behavior mediates the relationship between family strength and child behavior) was tested in this clinical sample. Instruments measuring family strength, parenting behavior, and child behavior were administered before, immediately after, and 6 months after participation in the SFP. To test the mechanisms of change, a half-longitudinal model was applied with two measurement points (before and after). A total of 62 families (50 boys, 24 girls, and 69 parents) participated in the culturally adapted SFP. Regarding the outcomes of the program, all variables yielded significant improvement in all variables. With respect to the mechanism of change, no significant association between the variables could be found. Implications for the implementation of the SFP in a clinical population and how further adaptation of the program could enhance the adherence of this target group are discussed.

## 1. Introduction

According to the latest available data from UNICEF [1], more than 13% of adolescents ages 10–19 years are estimated to live with a diagnosed mental disorder globally. Based on Bronfenbrenner’s [2] ecological systems theory, the microsystem (i.e., the closest context to a child/adolescent, such as family or kindergarten/school) has the greatest influence on the development of children. Therefore, interventions that take the family context into account are especially effective for reducing mental health problems in children/adolescents. The Strengthening Families Program (SFP) is a 14-session family group skills training program originally designed to help parents with substance use disorders to foster parenting skills, reduce family risk factors, and decrease problem behavior in children. It has been tailored to different age groups of children, from birth to 17 years of age; culturally adapted; and evaluated [3,4]. Between 2012 and 2018, nine courses of the SFP were implemented in the outpatient clinic of “Pro mente: kinder jugend familie” (pro mente: children youth families, herein pm:kijufa), which offers developmentally oriented diagnostics work as well as interventions and therapies for the individual child or for parents.

The first aim of the study was to evaluate outcomes regarding family strength, parenting behavior, and child behavior (e.g., reduction of externalizing problem behavior and enhancement of prosocial behavior and concentration). The second aim was to test the program’s mechanism of change as formulated by the program authors (i.e., to what extent parenting behavior mediates the relationship between family strength and behavior of the child).

### 1.1. Mental Health Problems in Children/Adolescents

There are two main categories of mental health disorders in children/adolescents: internalizing problem behavior and externalizing problem behavior [5]. A meta-analysis yielded that 13% of adolescents worldwide are affected by this symptomatology [6]. An Austrian study on mental health in teenagers (youth ages 10 to 18 years) found a point prevalence of 23.9% for any full-syndrome psychiatric disorder using the DSM-5 criteria [7]. Clinically relevant internalizing problems were reported more often (17.8%) than externalizing problems (7.4%). Furthermore, mental health disorders have a high comorbidity [7,8]. Over 40% of individuals within each diagnostic category met the criteria for another diagnostic category during their lifetime. The highest comorbidity rates were observed for aggression (86.7%) and anxiety/depression (86.5%) [7].

Concerning etiology, several studies have shown the interrelation between genetic and environmental influences on internalizing and externalizing problem behavior [9]. Regarding environmental influences, there is strong evidence that parenting behavior is related to internalizing and externalizing problem behavior in children [10,11] and can be either a risk or a protective factor for child developmental outcomes. Thus, preventions and interventions that foster effective parenting behavior, such as authoritative parenting and a warm and supportive family environment, have shown success in reducing mental health problems in children [12,13].

### 1.2. The Program Strengthening Families

**Figure 1:**
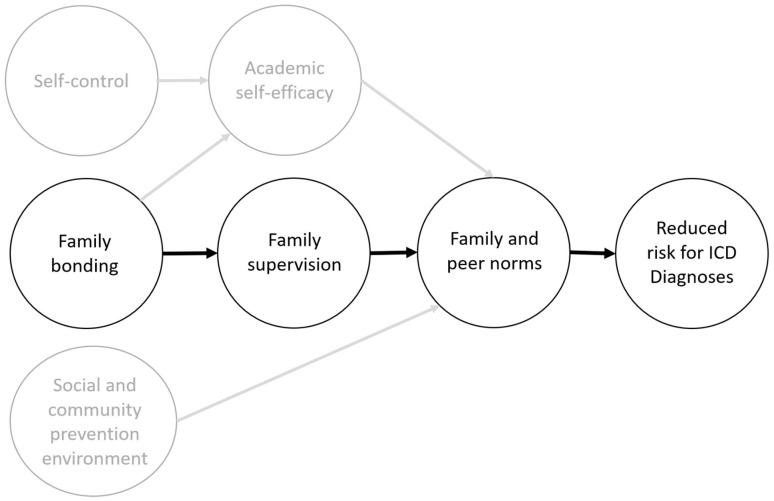
Mechanisms of change of the SFP [20] (p. 1764).

The SFP, developed in 1982 by Dr. Karol Kumpfer as part of a National Institute on Drug Abuse grant, was originally created to prevent substance abuse by strengthening family and parenting skills. It was nationally and internationally implemented and yielded positive outcomes not only in the reduction of substance abuse [14,15] but also for internalizing and externalizing problem behavior [16,17]. The SFP is based on two main theories: the family systems theory by Bowen [18] and the social learning theory by Ban-dura [19]. Applying the structural equation model, Kumpfer et al. [20] found that the pathway of family bonding, parental supervision, and communication of positive values and expectations was most predictive of a reduced risk for ICD diagnoses (Figure 1).

The program’s target group is parents and their children. It consists of 14 sessions, with 1 session per week. The structure of each session is 1 hour of training separately for parents and children conducted by one facilitator in each group. The session curricula for parents include content on goal setting, self-care, communication, relationships, setting limits, problem solving, and family management to prevent drug and alcohol use. The child session curricula include identifying goals and dreams; improving communication skills; identifying and coping with feelings; seeking support; problem solving; handling change; and undergoing psychoeducation on drugs, alcohol, and healthy lifestyle choices. After 1 hour, parents and children regroup and parent–child interactions, such as positive play and practicing skills, are fostered. Each session ends with a common meal. Furthermore, families get homework for the week (e.g., conducting family meetings). Each training session (i.e., parent, child, and family training) has a specific structure that is described in the manual. A detailed description of the SFP is described by Kumpfer et al. [21].

The SFP has been translated, culturally adapted, and implemented in 35 countries and is especially popular in Europe as its adaptation is relatively simple and does not require clinically trained staff for implementation [22]. An evaluation study of a German version of the SFP 10–14 years yielded no significant effects regarding substance use and parent-reported problem behavior [23]. However, an exploratory differential analysis of the German sample yielded that participants from high-risk groups achieved the best results compared with all other groups, especially for mental health and quality of life [24].

### 1.3. Implementation of the SFP in the Outpatient Clinic at Pm:kijufa

Pm:kijufa is a mid-sized community-based non-governmental organization operating in the Austrian state of Carinthia that provides different services for at-risk children/adolescents and their families. The main aim of pm:kijufa is to support children and adolescents with mental health problems and impaired social development. In doing so, pm:kijufa offers suitable services for this target group by taking into consideration not only medical (psychiatric) aspects but also psychological, social, and vocational factors. Furthermore, taking a socio-ecological perspective into account, family involvement is considered an important issue for all services, and pm:kijufa offers interventions covering parental support (for either biological or legal guardians). Pm:kijufa consists of four service domains: sociotherapy (consisting of crisis intervention centers and residential programs), vocational rehabilitation centers, prevention services, and two outpatient clinics. The clinics offer developmentally oriented diagnostics work in interdisciplinary teams as well as interventions and therapies for children/adolescents and their parents in individual or group settings.

The clinics especially care for children born at risk and/or with biological and/or psychosocial risks who later manifest developmental problems (emotional problems, conduct disorders, etc.). Each year, around 700 children and adolescents (*M*age = 9.2, *SD*age = 4; range: 2–18 years; 63% boys) and their families visit the outpatient clinics to receive developmentally oriented diagnostics and interventions. According to the Multiaxial Classification of Child and Adolescent Psychiatric Disorders system (MUAX) [25], around 40% of these children receive a diagnosis on Axis 1 (clinical psychiatric syndromes) and 45% on Axis 2 (specific disorders of psychological development). Within Axis 1, around 60% of patients are diagnosed with externalizing symptoms, such as hyperkinetic disorders (e.g., attention-deficit hyperactivity disorder) and conduct disorders. Within Axis 3 (intellectual level), 20% of children and adolescents are mentally impaired, and according to Axis 5 (associated abnormal psychosocial situations), 13% of children and adolescents face more than two abnormal life circumstances, especially in their immediate environment and upbringing. The global assessment of psychosocial disability (i.e., Axis 6 of the MUAX classification system) yields low psychological, social, and occupational functioning in 40% of children and adolescents. Furthermore, around two-thirds of children and adolescents exhibit comorbidities among Axes 1–4. Thus, the clinics offer multimodal treatment with single and group measurements conducted by psychologists, educationists, and occupational and speech therapists. As evaluation and quality assurance are central, most interventions are evidence based and their implementation and outcomes are proven on a regular basis.

Between 2012 and 2018, nine courses of the SFP for children ages 6–11 years were conducted at the pm:kijufa outpatient clinics in Carinthia (Austria). In sum, 20 employees of pm:kijufa, who were working as clinical psychologists or pedagogues, were trained in the SFP. For the first two courses, eight employees of pm:kijufa were trained by Karol Kumpfer and her colleagues. The other 12 employees were trained by another organization, which had a certificate in conducting SFP education for trainers. Each course was conducted by four trainers, two trainers for the parent training and two trainers for the child training. While the first two courses were monitored and evaluated by the program authors, the other courses were evaluated by Pro Mente Research, an independent institution responsible for quality assurance and evaluation for all service domains within the Pro Mente Group in Carinthia. The original manual was translated into German and slightly culturally adapted. The manual consists of three sections: parent training, child training, and family training. The cultural adaptation included changes of names, games, and songs. Concerning the implementation of the SFP in a clinical population, it was stated by the trainers that the group size is too big for the child group as children with severe conduct and social behavior disorders are difficult to handle by two trainers. Furthermore, the trainer mentioned that there were too many presentations that were too difficult for the children to understand and children with attention problems were not able to concentrate on the presentation for a longer period. Thus, more interactive games and more didactic elements should be implemented. To enhance participation and avoid attrition, transportation of the families to the outpatient clinic and childcare for younger children were provided.

### 1.4. The Present Study

Extending previous studies on evaluation of the SFP, the outcomes of the program in a clinical population (i.e., children with mental health disorders) were evaluated in a natural setting (i.e., the program and its evaluation were conducted without any supervision from research associates or the program developers). Although the SFP program has been implemented in many countries and its effectiveness has been proven, no evaluation study has been conducted in Austria with participants from a clinical population. Thus, we were interested in knowing whether there was an improvement in the outcome variables comparable to previous SFP studies.

Furthermore, understanding how an intervention produces its beneficial effects is essential for further program development and adaption. Parenting behavior was suggested as a potential mechanism of work by the program authors. They hypothesized that by increasing positive parenting, the program could contribute to several child behavior-related outcomes [20,21]. While parental monitoring and behavior management strategies were identified as critical components related to the positive effects of the SFP on substance abuse [26], evidence with respect to other desired outcomes of the SFP is missing.

The following hypotheses were tested:

**Hypothesis** **1** **(H1).** *After the intervention, better outcomes in all variables (i.e., improvement in family strength, parenting behavior, and children’s prosocial behavior and concentration and reduction in children’s aggressive behavior and hyperactivity) were expected.*

For the second set of hypotheses, we wanted to test how parenting behavior mediates the relationship between family strengths and children’s behavior outcomes.

**Hypothesis** **2a** **(H2a).** *Parenting behavior will mediate the relationship between family strengths and children’s aggressive behavior.*

**Hypothesis** **2b** **(H2b).** *Parenting behavior will mediate the relationship between family strengths and children’s hyperactivity behavior.*

**Hypothesis** **2c** **(H2c).** *Parenting behavior will mediate the relationship between family strengths and children’s prosocial behavior.*

**Hypothesis** **2d** **(H2d).** *Parenting behavior will mediate the relationship between family strengths and children’s concentration.*

## 2. Materials and Methods

### 2.1. Study Design and Procedure

All participants of the SFP were parents and their children who were clients of the pm:kijufa outpatient clinics in Carinthia. Families were chosen according to their need for a family-based intervention. The decision whether a family should take part was made by the clinical psychologists who diagnosed the child. Furthermore, only those families took part who were willing to participate at the training. Families with multiple and severe family problems, such as child neglect, child abuse, and violence, were not selected for this intervention.

Between 2012 and 2018, data were collected from parents and children at the beginning of (T0), end of (T1), and 6 months after (T2) the course. Participants were informed of the purpose and content of the questionnaire, and confidentiality was assured. Children were assisted by the trainers, while parents filled in the questionnaire independently under supervision of the course trainer. To compare participants on an individual level, the family level, and the group level, each person was given a code consisting of a common number for each family and individual numbers for each participant. Concerning children’s measures, most scales did not yield Cronbach’s alpha higher than 0.60, and thus these measures could not be used for the present study.

### 2.2. Study Sample

In sum, nine courses with 62 families (145 participants) were conducted. The whole sample consisted of 58 mothers, 8 fathers, 1 grandmother, 2 caregivers, and 74 children (24 girls). There were five courses in which at least one parent couple took part in the program together. However, mostly only mothers and their children attended the courses. The group size of each course ranged between four and nine families or 12 and 21 participants (see Table 1). The large variation in group sizes was due to high dropout rates in some courses, where more than five participants did not complete the program. The reasons for these high dropout rates were manifold (e.g., severe mental health problems among mothers, families who were overburdened by attending the program and fulfilling other duties for the family, and illness among children).

The characteristics of parents and families are displayed in Table 2.

Two-thirds of the children (69%) were boys, and the mean age was 9 years (range: 6–13). Concerning their diagnosis, around 80% of the children had an Axis 1 and/or an Axis 2 diagnosis. Around 40% had two or more diagnoses on Axis 1 or Axis 2 (comorbidities). Most diagnoses (80%) on Axis 1 were conduct or hyperkinetic disorders. Regarding Axis 2, 60% of the diagnoses were related to specific developmental disorders of speech and language (F80). With respect to Axis 5, half of the children faced two or more adversities, especially abnormalities, in their close environment. Finally, more than half of the children showed moderate to severe impairment in all domains and settings according to Axis 6 (see Table 3).

### 2.3. Measures and Instruments

Although measurements for parents and children were assessed, only measurements for parents were analyzed, as the scales for the children’s measurements yielded Cronbach’s alphas below 0.60 for reliability. Thus, only parents’ measures on children’s behavior were included in the analyses.

#### 2.3.1. Demographic Variables

Besides children’s and parents’ age and gender, several questions were asked about family characteristics, parents living with children, living conditions, and parents’ educational background.

#### 2.3.2. SFP Parent Interview Questionnaire

The standardized SFP Parent Interview Questionnaire (195 items) developed by Kumpfer et al. [27] was administered before (T0), immediately after (T1), and 6 months after (T2) the intervention. The 40-item parenting scale included subscales measuring parent supervision, parenting efficacy, SFP parenting skills, parental involvement, positive parenting, family organization, family communication, family conflict, and family strength/resilience.

#### 2.3.3. Parent Observation of Child Activities

The Parent Observation of Child Activities (POCA; scale originally developed by Kellam [28]) included subscales measuring children’s overt aggression, covert aggression, concentration problems, criminal behavior, impulsivity, hyperactivity, depression, and sociability.

As many of the original scales yielded reliabilities with Cronbach’s alphas less than 0.50, an exploratory factor analysis was conducted. Six scales that were slightly adapted from the original scales and yielded acceptable Cronbach’s alpha: family strength (α = 0.86), positive parenting (α = 0.77), children’s aggressive behavior (α = 0.73), hyperactivity (α = 0.77), prosocial behavior (α = 0.79), and concentration (α = 0.70) (see Table A1, Table A2, Table A3, Table A4, Table A5 and Table A6 in Appendix A).

### 2.4. Statistical Analyses

For statistical analyses, IBM SPSS Statistics Version 25.0 (IBM Corp., Armonk, NY, USA) and the lavaan package (version 0.6-9) for R statistics Version 4.0.4 (R Foundation for Statistical Computing, Vienna, Austria) [29,30] were employed. Families that did not complete the program were compared with families that did in terms of sociodemographic characteristics (e.g., educational status of parents, parental status, and the number and age of children); the chi-squared tests and *t*-tests (age and mean hours of work in a week) yielded no significant differences between these two groups (see Table 2). Furthermore, no differences were found for parents’ measures between these two groups from the first point of measurement (see Table 4). Thus, only parents who completed the program were included for further analysis. To handle missing data for participants who completed the program but did not provide data at T1, full information maximum likelihood (“missing = ML”) was used [31].

To measure the outcomes of the program, paired *t*-tests were applied for each outcome variable between T0 and T1, T0 and T2, and T1 and T2. This analytical approach was chosen due to high data attrition at T2, which would have resulted in a strong reduction in the sample if using a repeated analysis of variance.

**Figure 2:**
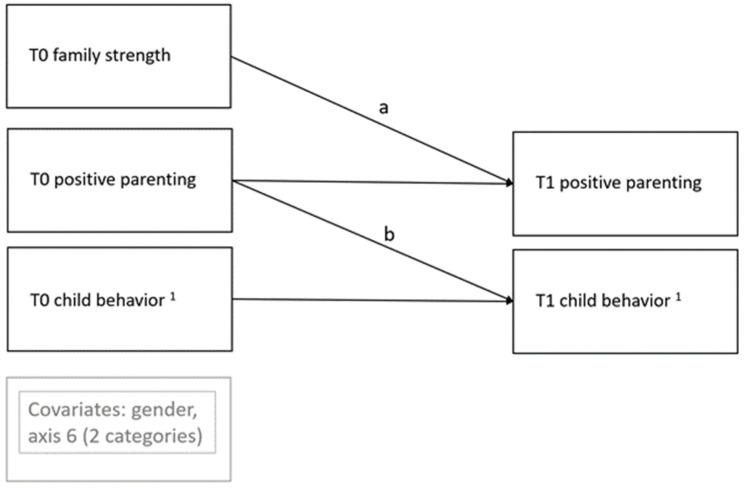
Graphical illustration of the half-longitudinal model. ^1^ aggressive behavior, hyperactivity, prosocial behavior, child concentration.

To test the program’s mechanism of change, a half-longitudinal model proposed by Cole and Maxwell [32] for testing mediation with two time points was used. This model is an auto-regressive, cross-lagged path model. In sum, four models were separately tested for each child behavioral outcome (i.e., aggressive behavior, hyperactivity, prosocial behavior, and concentration). To test the indirect or mediation effect (Path a × b) on the child behavioral outcome, Path a of the model was tested by regressing T1 parents’ positive parenting on T0 family strength. Path b of the model was tested by regressing T1 child behavioral outcome on T0 positive parenting. A simplified graphical illustration of the half-longitudinal model is shown in Figure 2.

Child gender and Axis 6 were included as dichotomized variables (0 = no impairment; 1 = moderate to severe impairment) in all four models. Goodness-of-fit was evaluated using the chi-squared (χ^2^) test of model fit (χ^2^/*df)*, the comparative fit index (CFI), the standardized root mean square residual (SRMR), and the root mean square error of approximation (RMSEA). According to conventional guidelines, a non-significant χ^2^, χ^2^/*df* values less than 3, CFI indices greater than 0.95, and SRMR and RMSEA values less than 0.008 indicate an acceptable model fit [33]. The significance of these indirect effects (i.e., the product of the Paths a and b in the mediation model) was tested by bootstrapping the 95% confidence interval of the indirect effect using Selig and Preachers’ [34] online Monte Carlo simulation calculator with 1000 repetitions. A confidence interval that did not include 0 provided evidence for a significant indirect effect.

## 3. Results

### 3.1. Change in Outcomes after Completion of the SFP

To test our first hypothesis, several two-tailed *t*-tests were run to compare the means of family strength, positive parenting, and children’s behavior at T0 and T1. Additionally, effect sizes of the outcomes were calculated using Cohen’s *d*. As there was no control group, the effect sizes of the pm:kijufa sample were compared with the effect sizes for the SFP 6–11 years reported by Kumpfer [27]. (Table 5).

Neither a significant increase nor a decrease between T1 and T2 was found for any of these variables.

### 3.2. The SFP’s Mechanism of Change

Results of the half-longitudinal model analyzing the association between family strength, positive parenting behavior, and child aggressive behavior are presented in Table 6. The model showed a good fit: χ^2^ (*df* = 6) = 4.776, *p* = 0.573, χ^2^/*df* = 0.796, CFI = 1, SRMR = 0.042, and RMSEA = 0.00. No significant relationship was found either for family strength at T0 with positive parenting behavior at T1 or for positive parenting at T0 with child aggressive behavior at T1 (controlling for gender and Axis 6). The indirect effect of family strength at T0 on child aggressive behavior at T1 yielded no significant association. As the 95% bootstrapped CI of the indirect effect included 0, the indirect effect was not significant.

Results of the half-longitudinal model analyzing the association between family strength, positive parenting behavior, and child hyperactivity are presented in Table 7. The model showed a good fit: χ^2^ (*df* = 6) = 7.447, *p* = 0.281, χ^2^/*df* = 1.241, CFI = 0.983, SRMR = 0.052, and RMSEA = 0.053. Family strength at T0 was not significantly associated with positive parenting behavior at T1. However, positive parenting at T0 was positively associated with child hyperactivity at T1 (controlling for gender and Axis 6). The indirect effect of family strength at T0 on child hyperactivity at T1 yielded no significant association. As the 95% bootstrapped CI of the indirect effect included 0, it must be assumed that the indirect effect was not significant.

Results of the half-longitudinal model analyzing the association between family strength, positive parenting behavior, and child prosocial behavior are presented in Table 8. The model showed a good fit: χ^2^ (*df* = 6) = 7.159, *p* = 0.306, χ^2^/*df* = 1.193, CFI = 0.989, SRMR = 0.051, and RMSEA = 0.048. Family strength at T0 was not significantly associated with positive parenting behavior at T1, nor was positive parenting at T0 associated with child prosocial behavior at T1 (controlling for gender and Axis 6). The indirect effect on family strength at T0 on child prosocial behavior at T1 yielded no significant association. As the 95% bootstrapped CI of the indirect effect included 0, it must be assumed that the indirect effect was not significant.

Results of the half-longitudinal model analyzing the association between family strength, positive parenting behavior, and child concentration are presented in Table 9. The model showed a good fit: χ^2^ (*df* = 6) = 6.745, *p* = 0.345, χ^2^/*df* = 1.124, CFI = 0.991, SRMR = 0.061, and RMSEA = 0.038. Family strength at T0 was not significantly associated with positive parenting behavior at T1, nor was positive parenting at T0 associated with child concentration at T1 (controlling for gender and Axis 6). The indirect effect of family strength at T0 on child concentration at T1 yielded no significant association. As the 95% bootstrapped CI of the indirect effect included 0, the indirect effect was not significant.

## 4. Discussion

Mental health problems in children and youth are a major concern for the whole society. As families have the greatest influence on children’s well-being, it is necessary to provide interventions not only for children but also for their parents or caregivers. Programs such as the SFP were developed for the whole family to change several factors that are influential in children’s development, such as parenting behaviors and family communication. Many clients from the two outpatient clinics of pm:kijufa are affected by mental health problems, with most of them externalizing problem behavior. To include families in the children’s therapy, the SFP was implemented. During the first two years, the program developer Karin Kumpfer and colleagues monitored and evaluated the program; after that, it was evaluated by an independent research unit of pro mente.

The goals of the present study were twofold: first, we investigated whether the pro-gram was effective in fostering family strength and positive parenting behavior and im-proving child behavior (reducing aggressive behavior and hyperactivity and enhancing prosocial behavior and concentration). Secondly, applying a half-longitudinal model, we tested the mechanisms of change as formulated by the program authors.

In line with the first hypothesis and with the results of previous studies (e.g., [22,27]), there was a significant improvement in parenting behavior, family strength, and children’s prosocial behavior and concentration. Furthermore, children’s aggressive behavior and hyperactivity significantly decreased between pre- and post-test. These results lend support to the notion that the program is effective in a clinical population in a natural setting without supervision from research staff or the program developer. However, due to limited resources and for ethical reasons, it was not possible to provide control groups, as the staff from the outpatient clinics selected those families that were at high risk and were willing to participate in the program. Therefore, the effect size was also taken into consideration and compared with effect sizes yielded from an evaluation study with over 1600 families between 2004 and 2007 [27]. The overall effect sizes of all measured scales showed medium effects. While effect sizes for family strength, positive parenting, and child concentration were lower compared to Kumpfer [27], they were similar for child aggression and prosocial behavior and higher for child hyperactivity. Furthermore, results from an SFP evaluation study in Germany showed that children with elevated psychosocial risk benefited most from participation in the SFP with respect to mental health [24]. As the pm:kijufa sample consisted of clients with at least one mental health diagnosis who were from high-risk families, it can be assumed that participation in the SFP was beneficial for these clients.

Results of the half-longitudinal model analyzing the association between family strength, positive parenting, and children’s behavior showed no significant relationships between the variables, except for the significant path between parenting behavior and child hyperactivity. Thus, the theoretical model suggested by Kumpfer et al. [20] was not supported in the pm:kijufa sample. Although the scales were slightly changed and not all scales of the model from Kumpfer et al. [20] were used, the theoretical assumptions are in line with their model and should have led to significant associations. Furthermore, as this was the first study to test the longitudinal associations of family strength, parenting behavior, and children’s behavior, these analyses were exploratory. Mediation analyses in the current study might be underpowered [35], and more empirical studies as well as theoretical considerations are needed as a foundation to provide high-quality evidence for the SFP’s potential mechanisms of work.

Neither a direct nor an indirect effect could be found between parenting behavior and children’s behavior, unlike in previous studies [11]. Thus, it is not clear which components of the program led to children’s behavioral outcomes. As the program has separate sessions for parents and children, it can be assumed that the specific training sessions for parents and children were related to the outcome rather than the parenting behavior. Thus, future studies are needed to investigate the program’s mechanism of change.

Around 30% of the participants did not finish the program and dropped out after the first two sessions. However, the courses differed in terms of the dropout rate: in five out of nine SFP courses, more than one-third of the participants dropped out of the program. Only some participants provided a reason for why they could not finish the program. However, many participants were not available after their dropout and could not be contacted. Although some previous studies have shown that children with behavioral difficulties, such as conduct disorders, are more likely to drop out [36], this was not the case in the pm:kijufa sample. Thus, in future studies, more effort must be made to contact these participants to find out why they left the program [37].

Concerning participants, the SFP is intended for the whole family to take part in the program. However, participants were mostly mothers and their children and only in some courses the whole family participated. This participation pattern was similar to that of several other SFP courses (e.g., Baldus [23] had 141 mothers and 5 fathers; more than two-thirds of the participants were female in the study by Kumpfer [27]). In Western societies, the traditional gender role of mothers as being mainly responsible for parenting and educational tasks is still common. However, it is necessary to adapt the program to make it more accessible for the whole family. One possibility could be to implement a short version of the program, as was done in a Spanish sample [16], which yielded significant positive results with only six sessions. Thus, future studies could implement short and long versions of the program at the same time and compare their effectiveness.

Another important issue is that the internal consistency of the original scales created by the program developers could not be verified in our clinical population. Especially regarding instruments for children’s/adolescents’ self-assessment, no reliable scales could be found. For the parents’ measures, slightly adapted scales with satisfactory Cronbach’s alpha were used, but for children and adolescents, it was not possible to form reliable scales. This modification might limit the comparability of the study.

## 5. Conclusions

The results of the program evaluation showed an increase in family strength, positive parenting behavior, and child prosocial behavior and concentration as well as a reduction in externalizing problem behavior in children. Although it was not possible to conduct a study with a control group due to ethical and financial reasons, the results confirm the external validity of the program, as it was conducted without any influence from the pro-gram authors. Our findings did not mirror earlier research regarding positive parenting as a working mechanism of the program, and more research to examine the mechanisms of the program is necessary. Despite this limitation, programs such as the SFP should be implemented on a regular basis in normal and clinical populations, as they contribute to healthy families and children’s well-being.

## Figures and Tables

**Table 1:** Courses and participants.

Participants (*n*)	A	B	C	D	E	F	G	H	I	SUM
Mother/father	9/1	8/0	8/2	6/1	4/0	5/0	6/1	6/3	9 */0	69
Boy/girl	9/2	7/2	8/2	6/2	4/4	3/4	4/1	5/4	6/3	74
Families	9	8	8	6	4	5	6	7	9	62
Total	21	17	20	15	12	12	12	18	18	145
Dropouts (*n*)	2	9	2	9	10	9	8	4	0	53
Dropout rate	Low	High	Low	High	High	High	High	Low	Low	

* grandmother (*n* = 1); caregivers (*n* = 2).

**Table 2:** Characteristics of parents and families.

Characteristic	Participants	Dropouts	*df*	χ^2^	*p*
Female gender (%)	89.4%	94.7%	1	0.49	0.482
Age, *Mean* (*SD*)	35.84 (6.45)	35.37 (5.99)			0.454
Number of work hours per week, *Mean* (*SD*)	27.73 (11.18)	31.93 (7.94)			0.349
Education level (*n*)					
Elementary school or less	9	3	2	1.74	0.419
Middle school	31	7			
High school or higher	14	1			
Number of children (*n*)					
1	9	3	3	0.77	0.855
2	23	4			
3	16	3			
More than 3	7	1			
Parents living with child (*n*)					
Both parents	36	11	2	0.16	0.922
Single mother/father	23	8			
Other: grandparents, foster care	3	1			
Living situation (*n*)					
Rented apartment	33	7	2	0.89	0.645
Owner-occupied flat/house	27	5			
Foster care flat	3	0			

**Table 3:** Characteristics of children.

Sociodemographics	Participants
Female gender (%)	31%
Age, *Mean* (*SD*)	8.79 (1.69)
MUAX Diagnosis	*n*
Axis 1: Clinical psychiatric syndromes	62
Hyperkinetic disorders (F90, F90.0, F90.8, F90.9)	23
Hyperkinetic conduct disorders (F90.1)	11
Conduct disorders (F91)	20
Mixed disorders of conduct and emotions (F92)	9
Other diagnoses (e.g., other behavioral and emotional disorders with onset usually occurring in childhood or adolescence, F98)	16
Axis 2: Specific disorders of development	36
Specific developmental disorders of speech and language (F80)	33
Specific developmental disorders of scholastic skills (F81)	9
Specific developmental disorders of motor function (F82)	13
Axis 5: Associated abnormal psychosocial conditions	
Children/adolescents with two or more adversities	39
Axis 6: Global social functioning	
Moderate to severe impairment in all domains and settings	44

**Table 4:** *t*-test results: participants vs. dropouts at T0 for the studied variables.

Characteristic	Participants			Dropouts			*p*
	*n*	*M*	*SD*	*n*	*M*	*SD*	
Family strength	63	3.59	0.69	21	3.55	0.87	0.793
Positive parenting	68	4.05	0.53	22	4.25	0.41	0.119
Child aggression	67	1.86	0.35	23	1.79	0.49	0.407
Child hyperactivity	68	2.66	0.69	22	2.55	0.87	0.566
Child prosocial behavior	65	3.37	0.60	21	3.39	0.64	0.859
Child concentration	67	2.98	0.79	21	3.09	0.81	0.596

**Table 5:** *t*-test results and effect sizes for differences in the studied variables between T0 and T1.

Characteristic	*N*	*M*_T0_	*SD*	*M*_T1_	*SD*	*M*_diff_	*T*	*p*	*ES*_d_ ^1^	*ES*_dKumpfer_ ^2^
Family strength	47	3.68	0.67	3.95	0.70	−0.27	−3.56	0.00	0.39	0.76
Positive parenting	52	4.10	0.53	4.33	0.46	−0.23	−4.31	0.00	0.50	0.67
Child aggression	51	1.91	0.37	1.75	0.35	0.15	3.08	0.00	0.43	0.46
Child hyperactivity	51	2.7	0.62	2.43	0.59	0.27	2.88	0.01	0.46	0.03
Child prosocial behavior	48	3.37	0.59	3.57	0.65	−0.20	−2.80	0.01	0.31	0.32
Child concentration	51	2.99	0.79	3.26	0.74	−0.27	−2.19	0.03	0.36	0.59

^1^ Cohen’s *d*; ^2^ Cohen’s *d* reported by Kumpfer [27]. Note: For aggression and hyperactivity, lower values are better.

**Table 6:** Paths between family strength, positive parenting, and child aggression.

Paths	*B*	*SE*	*p*	95% CI
Autoregressive paths				
T0 FS → T1 FS	0.683	0.113	<0.001	[0.495, 0.929]
T0 PP → T1 PP	0.602	0.106	<0.001	[0.383, 0.809]
T0 AG → T1 AG	0.502	0.124	<0.001	[0.319, 0.793]
Paths for indirect effects				
T0 FS → T1 PP (a)	−0.056	0.081	0.486	[−0.203, 0.120]
T0 PP → T1 AG (b)	0.118	0.116	0.309	[−0.110, 0.344]
Indirect effect	−0.007	0.014	0.641	[−0.036, 0.024]
Covariates				
Gender → T1 PP	0.057	0.150	0.704	[−0.232, 0.341]
Gender → T1 AG	−0.053	0.110	0.631	[−0.268, 0.172]
Axis 6 → T1 PP	−0.071	0.103	0.490	[−0.267, 0.143]
Axis 6 → T1 AG	0.048	0.086	0.574	[−0.205, 0.119]

Note: FS, family strength; PP, positive parenting; AG, aggression; gender (0 = girl, 1 = boy); Axis 6 (0 = superior, adequate, or slight impairment; 1 = moderate to severe impairment).

**Table 7:** Paths between family strength, positive parenting, and child hyperactivity.

Paths	*B*	*SE*	*p*	95% CI
Autoregressive paths				
T0 FS → T1 FS	0.665	0.103	<0.001	[0.485, 0.895]
T0 PP → T1 PP	0.596	0.113	<0.001	[0.369, 0.810]
T0 HY → T1 HY	0.406	0.133	0.002	[0.160, 0.677]
Paths for indirect effects				
T0 FS → T1 PP (a)	−0.055	0.081	0.495	[−0.196, 0.129]
T0 PP → T1 HY (b)	0.382	0.197	0.052	[−0.044, 0.764]
Indirect effect	−0.021	0.034	0.537	[−0.094, 0.047]
Covariates				
Gender → T1 PP	0.053	0.149	0.723	[−0.232, 0.354]
Gender → T1 HY	0.213	0.168	0.203	[−0.126, 0.546]
Axis 6 → T1 PP	−0.066	0.104	0.525	[−0.249, 0.154]
Axis 6 → T1 HY	0.185	0.138	0.180	[−0.101, 0.454]

Note: HY, hyperactivity; gender (0 = girl, 1 = boy); Axis 6 (0 = superior, adequate, or slight impairment; 1 = moderate to severe impairment).

**Table 8:** Paths between family strength, positive parenting, and child prosocial behavior.

Paths	*B*	*SE*	*p*	95% CI
Autoregressive paths				
T0 FS → T1 FS	0.665	0.103	<0.001	[0.485, 0.895]
T0 PP → T1 PP	0.596	0.113	<0.001	[0.369, 0.810]
T0 PS → T1 PB	0.406	0.133	0.002	[0.160, 0.677]
Paths for indirect effects				
T0 FS → T1 PP (a)	−0.053	0.082	0.518	[−0.202, 0.126]
T0 PP → T1 PB (b)	−0.033	0.167	0.842	[−0.382, 0.285]
Indirect effect	0.002	0.016	0.913	[−0.035, 0.028]
Covariates				
Gender → T1 PP	0.058	0.150	0.697	[−0.232, 0.351]
Gender → T1 PB	−0.203	0.202	0.315	[−0.593, 0.207]
Axis 6 → T1 PP	−0.057	0.106	0.594	[−0.245, 0.171]
Axis 6 → T1 PB	0.005	0.157	0.979	[−0.293, 0.306]

Note: PB, prosocial behavior; gender (0 = girl, 1 = boy); Axis 6 (0 = superior, adequate, or slight impairment; 1 = moderate to severe impairment).

**Table 9:** Paths between family strength, positive parenting, and child concentration.

Paths	*B*	*SE*	*p*	95% CI
Autoregressive paths				
T0 FS → T1 FS	0.642	0.112	<0.001	[0.458, 0.892]
T0 PP → T1 PP	0.608	0.109	<0.001	[0.382, 0.830]
T0 CON → T1 CON	0.275	0.138	0.046	[−0.013, 0.533]
Paths for indirect effects				
T0 FS → T1 PP (a)	−0.066	0.082	0.422	[−0.205, 0.127]
T0 PP → T1 CON (b)	0.098	0.197	0.499	[−0.262, 0.514]
Indirect effect	−0.006	0.022	0.768	[−0.063, 0.030]
Covariates				
Gender → T1 PP	0.065	0.149	0.661	[−0.229, 0.357]
Gender → T1 CON	−0.278	0.246	0.259	[−0.752, 0.214]
Axis 6 → T1 PP	−0.061	0.106	0.566	[−0.245, 0.170]
Axis 6 → T1 CON	−0.108	0.179	0.546	[−0.452, 0.241]

Note: CON, concentration; gender (0 = girl, 1 = boy); Axis 6 (0 = superior, adequate, or slight impairment; 1 = moderate to severe impairment).

## Data Availability

The data that support the findings of this study are available from the corresponding author, [E.S.], upon reasonable request.

## Appendix A

**Table A1:** Positive parenting.

Par001: I praise my child when he/she has behaved well
Par002: I use clear directions with my child
Par013: I follow through with reasonable consequences when rules are broken
Par014: I reward completed chores with allowances or privileges
Par015: I talk to my child about his or her plans for the next day or week
Par016: I talk to my child about his or her friends
Par017: I know where my child is and who he/she is with
Par018: I talk to my child about his/her feelings
Par019: I use time outs when my child will not do what I ask
Par022: I talk to my child about how he/she is doing in school
Par023: I check to see if my child completes his/her homework

**Table A2:** Family strength.

fam01: Family Supportiveness/Love/Care
fam04: Family Organization (rule, chore, self-responsibility)
fam06: Family Unity (togetherness, cohesion)
fam07: Positive Mental Health
fam08: Physical Health
fam09: Emotional Strength
fam10: Knowledge and Education
fam11: Social Networking (friends, community)
fam12: Spiritual Strength

**Table A3:** Child concentration.

Poca01: Completes work and chores
Poca04: Concentrates
Poca20: Is eager to learn
Poca28: Stays on task until completed

**Table A4:** Overt, covert aggression.

Poca03: Is stubborn
Poca05: Breaks rules
Poca17: Takes others’ property
Poca19: Fights
Poca21: Damages other’s property on purpose
Poca23: Lies
Poca25: Argues with adults
Poca27: Teases other children
Poca30: Skips school
Poca31: Uses a weapon in a fight
Poca35: Starts physical fights with other children
Poca39: Loses temper

**Table A5:** Prosocial behavior.

fam01: Family Supportiveness/Love/Care
fam04: Family Organization (rule, chore, self-responsibility)
fam06: Family Unity (togetherness, cohesion)
fam07: Positive Mental Health
fam08: Physical Health
fam09: Emotional Strength
fam10: Knowledge and Education
fam11: Social Networking (friends, community)
fam12: Spiritual Strength

**Table A6:** Hyperactivity.

Poca29: Can’t sit still
Poca33: Runs around a lot, climbing on things
Poca37: Is always “on the go”
